# New prefilled syringe aflibercept design. A cause of symptomatic IOP spike after aflibercept PFS?

**DOI:** 10.22336/rjo.2024.41

**Published:** 2024

**Authors:** Irene Loscos-Giménez, Daniela Rego-Lorca, Francisca Bassaganyas-Vilarrasa, Jaume Crespí-Vilimelis, Jesús Díaz-Cascajosa, José Ignacio Vela Segarra

**Affiliations:** 1 Department of Ophthalmology, Hospital de la Santa Creu i Sant Pau, Barcelona, Spain; 2 Institut d´Investigació Biomèdica Sant Pau (IIB SANT PAU), Barcelona, Spain; 3 Universitat Autònoma de Barcelona, Bellaterra, Spain

**Keywords:** Aflibercept, injection, prefilled, syringe, intraocular pressure, PSF = prefilled syringes, IVI = intravitreal injection, AMD = age-related macular degeneration, DME = diabetic macular edema, RVO = retinal vein occlusion, IOP = increased intraocular pressure, FDA = Food and Drug Administration, FBV = medical retina department, VA = visual acuity, GAT = Goldmann Applanation Tonometer, VPP = pars plana vitrectomy, EMA = European Medicines Agency

## Abstract

**Objective:**

To describe ocular hypertension cases after using a new aflibercept prefilled syringe and to assess the main characteristics of these eyes and their possible association with intraocular pressure (IOP) changes.

**Methods:**

Case series. We reported all the cases of ocular hypertension following aflibercept prefilled syringes (PFS) treatment in our department between April 2021 and December 2023.

**Results:**

A total of 4183 eyes were treated with aflibercept PSF. Thirteen transitory IOP elevations were observed immediately after injection (0.3%). Two eyes had an IOP between 30-35 mmHg, five eyes had an IOP between 36-55 mmHg and three eyes had an IOP > 56 mmHg. The mean IOP was 45.5 mmHg±11.33. Only six eyes needed anterior chamber paracentesis (37.5%). The other patients were treated conservatively (ocular massage and/or IOP-lowering drops). The mean IOP after treatment was 15.71 mmHg±7.20. Visual acuity improved after treatment in all the patients.

**Discussion:**

Compared with other injectors, reports have indicated a higher incidence of moderate and severe IOP spikes with aflibercept PSF. The European Medicine Agency (EMA) has associated this significant increase with incorrect syringe handling, leading to higher injection volumes. Although plunger misalignment seems to play a role in the IOP spikes, some other characteristics of this new injector could play a role. Factors such as syringe diameter, plunger alignment, and injection force may contribute to this issue. The reason some authors found no significant differences in IOP elevation after IVI, with aflibercept PFS, could be due to variations in patient characteristics, which may also play an important role in post-IVI pressure changes.

**Conclusions:**

Intraocular pressure spikes after aflibercept PFS can be explained by injector characteristics. The PFS of aflibercept has a domed plunger. Incorrect alignment between the base of the plunger and the black dosing line could cause an increase in the injected volume. Furthermore, the wider syringe diameter of aflibercept PFS could imply a larger injection force, increasing the risk of IOP elevation. Patient characteristics, such as previous VPP, axial length, or glaucoma history, may also play a role. Further studies are required to develop an ideal intravitreal syringe.

## Introduction

Intravitreal therapy has changed the prognosis of many retinal disorders such as exudative age-related macular degeneration (AMD), diabetic macular edema (DME), diabetic retinopathy, retinal vein occlusion (RVO), and myopic choroidal neovascularization [1-3]. Although it is usually a safe outpatient procedure, there is a risk of serious complications such as vitreous hemorrhage, traumatic cataract, retinal detachment, endophthalmitis, or increased intraocular pressure (IOP) [1,2]. The latter has been reported both as a hypertensive spike immediately after intravitreal injection (IVI) and as a persistent increase in IOP secondary to repeated IVI [1-4]. In this study, we focused on the IOP increase immediately post-injection, and this has also been described as a contributing factor to the long-term development of sustained IOP elevation [4].

In 2019, the Food and Drug Administration (FDA) approved the use of aflibercept (Eylea®; Bayer, Inc.)-prefilled syringes (PFS) for intravitreal administration. In April 2021, Bayer reported an increase in IOP immediately after the injection when using this PFS. They reported an increase in the incidence of IOP elevation following IVI from 0.15 cases to 1.1 cases per 10,000 injections when aflibercept PFS was used [5]. Other authors have also reported higher rates of IOP elevation after IVI since the marketing of aflibercept PFS [6,7].

The retinal medical department has noted an increased incidence of severe transient vision loss and larger injection forces with aflibercept PFS adoption compared with traditional aflibercept vial preparations. We decided to study the possible association with IOP spikes after aflibercept PFS and assess the main characteristics of these eyes.

## Results

This case series study included consecutive patients who presented ocular hypertension after the treatment with aflibercept PFS IVI for exudative AMD, DME, or RVO-associated macular edema in our Ophthalmology Department, between April 2021 and December 2022.

Intravitreal injection (IVI) was performed by attending the medical retina department (FBV). All injections were administered under topical anesthesia (lidocaine 20 mg/g ophthalmic gel), and before the injection, eyes were treated with chlorhexidine 0.1% drops applied to the conjunctiva. Injections were performed 3.5-4.0 mm posterior to the corneoscleral limbus in the inferotemporal quadrant using a 30-gauge needle, with the patient lying supine on a medical couch. The injected volume was 0.5 ml in all cases. No ocular massage or anterior chamber paracentesis was performed before the injection. Hand motion VA was verified immediately after injection.

The physician performing the injections was asked to report any episodes of significant vision loss or suspected IOP spike immediately after aflibercept PFS IVI. IOP was measured using a Goldmann Applanation Tonometer (GAT), the visual complaint was recorded as blurred vision or NLP, and the physician who performed the injection was registered as an assistant or resident. Treatment was initiated with ocular massage, IOP-lowering drops, or anterior chamber paracentesis. IOP after treatment was also recorded using the GAT. IOP was not registered in patients without visual loss symptoms as in our regular practice.

The medical records of the included patients were reviewed, and age, sex, underlying diagnosis, lens status, axial length, prior ocular surgeries, history of glaucoma or ocular hypertension, use of IOP-lowering drops, previous IOP spike episodes, and number of previous IVI were recorded.

Between April 2021 and December 2022, 6962 eyes were treated with IVI in our Ophthalmology Department. Of them, 4186 eyes received aflibercept PFS. Thirteen transitory symptomatic IOP elevations were observed immediately after the aflibercept PFS injection (0.31%). Eight of these patients experienced blurred vision, and five had NLP after injection. During the first year, eight IOP transitory elevations were observed, decreasing the incidence during the following months.

A chart review was performed for the 13 patients (**Table 1,2**).

**Table 1:** Chart review for the 13 patients (BV = Blurred vision, NLP = Non-light perception, NA = Not available, AMD = age-related macular degeneration, DME = diabetic macular edema, RVO = retinal vein occlusion)

Patient	Sex	Age	Previous diagnosis	Treated eye	IOP	IOP post-treatment	IVI Visual acuity	Acuity post-treatment
1	Woman	85	AMD	Left	40	8	BV	Improved
2	Man	73	DME	Right	NA	NA	BV	Improved
3	Man	74	AMD	Right	NA	NA	NLP	Improved
4	Woman	87	AMD	Right	40	NA	NLP	Improved
5	Woman	31	DME	Left	42	12	BV	Improved
6	Man	57	DME	Left	58	13	NLP	Improved
7	Man	77	AMD	Right	40	10	BV	Improved
8	Man	65	DME	Left	30	19	BV	Improved
9	Man	93	AMD	Right	30	NA	NLP	Improved
10	Woman	63	AMD	Right	NA	NA	BV	Improved
11	Woman	52	DME	Left	35	29	BV	Improved
12	Man	68	RVO	Right	60	NA	BV	Improved
13	Woman	85	AMD	Left	50	19	NLP	Improved

**Table 2:** Treatment and history information (NA = Not available, ACP = Anterior chamber paracentesis, OM = Ocular massage, LD = Lowering drops)

Patient	Treatment Performance	IVI	Glaucoma history	IOP elevation	PSF	Nº previous IVI	Lowering drops	Previous VPP	Axial lengths
1	ACP	Aflibercept PSF	No	No	No	24	N2	No	22.22
2	ACP	Aflibercept PSF	No	No	Yes	20	No	No	NA
3	OM + LD	Aflibercept PSF	No	No	NA	1	No	No	NA
4	ACP	Aflibercept PSF	No	No	Yes	38	No	No	NA
5	OM+LD	Aflibercept PSF	No	No	No	39	No	No	NA
6	ACP	Aflibercept PSF	No	No	No	16	Yes	Waiting list	NA
7	ACP	Aflibercept PSF	Yes	Yes (ranizumab)	No	29	Yes	No	23.88
8	OM	Aflibercept PSF	No	No	No	6	No	Yes	NA
9	ACP	Aflibercept PSF	No	No	No	31	No	No	NA
10	LD	Aflibercept PSF	No	No	No	21	No	No	NA
11	OM+LD	Aflibercept PSF	No	No	No	4	No	Improved	NA
12	OM	Aflibercept PSF	No	No	Yes	19	No	Improved	24.35
13	OM + LD	Aflibercept PSF	Yes	No	Yes	9	No	Improved	22.37

The mean age was 70±16.9 years old (range was 31-93 years). Seven patients (69.2%) were men. The indications for aflibercept treatment were AMD (n=7), DME (n=5), or RVO (n=1). One patient had a history of pars plana vitrectomy (VPP) for endophthalmitis after dexamethasone implant injection. One patient had a vitreous hemorrhage. Two patients were treated for glaucoma (n=1) or ocular hypertension (n=1). One patient had a history of acute glaucoma in an untreated eye. Four eyes (30.7%) were found to be pseudophakic. The average axial length of the sample was 23.2±1.07. The mean number of previous IVIs was 23±12.4.

The IOP was measured immediately after the procedure. Two eyes had an IOP between 30-35 mmHg, five eyes had an IOP between 36-55 mmHg and three eyes had an IOP > 56 mmHg. The mean IOP was 45.5 mmHg±11.3.

Anterior chamber paracentesis was performed in six eyes (37.5%), all from patients who referred NLP after the injection. Seven eyes (53.8%) were managed conservatively: two with ocular massage (15.4%), one with IOP-lowering drops (7.7%), and four with a combination of IOP-lowering drops and ocular massage (30.8%). Five minutes after treatment, IOP was measured using Goldman tonometry. The mean IOP after treatment was 15.7 mmHg±7.2. Visual acuity improved after treatment in all patients.

## Discussion

A higher incidence of moderate and severe IOP elevation has been reported after IVI when aflibercept PFS was used [6-9]. European Medicines Agency (EMA) notified an increment of sevenfold with aflibercept PFS, going from 0.15 cases every 10.000 vials to 1.1 cases every 10.000 PFS [5]. We report 13 cases, representing 3.10 cases for every 10.000 IOP elevation following IVI with aflibercept PFS. Because of this increase, we have been forced to analyze the characteristics of these eyes and the possible predisposing factors.

According to the EMA, IOP spikes after IVI with aflibercept PFS could be explained by the incorrect handling of aflibercept PFS [5]. Compared with other injectors (tuberculin syringe with a 30G needle or ranibizumab PFS), aflibercept PFS has a domed plunger. An incorrect alignment between the base of the plunger and the black dosing line could cause an increased volume administration to the intravitreal space. Guest et al. [10] reported that an incorrect plunger position can cause an IVI volume 71% higher in aflibercept PFS than in the BD Luer-Lok 1-ml syringe. In addition, the internal diameter of aflibercept PFS was 6.4 mm, compared to 4.6 mm for ranibizumab PFS. Consequently, a small variation of the plunger position had an important effect on the volume of the drug when aflibercept PFS was used [6,10,11].

Although plunger misalignment seems to play a role in the transitory IOP rise observed post-IVI, some other characteristics of this new injector could play a role. According to our experience, a higher injection force is applied when using aflibercept PFS than when using Lucentis PFS. Lee et al. [7] suggested that the wider syringe diameter of aflibercept PFS implies a larger injection force, even if the plunger is aligned painstakingly. Moreover, when reviewing the literature, another factor that has been associated with IOP rise after IVI is the use of small-gauge needles due to less vitreous reflux [4]. In our study, all patients were injected using a 30-gauge needle, as recommended by the EMA guidelines.

Despite this, controversy remains as to whether aflibercept PFS is associated with an increased incidence of elevated IOP after IVI. Dingerkurs et al. [8] did not find significant differences in IOP elevation after IVI with aflibercept PFS [8,9]. These discrepancies in different studies could be due to variations in patient characteristics, which may also play an important role in post-IVI pressure changes.

One relevant patient characteristic may be the total number of previous IVIs. Some observational studies have reported an incremental risk of sustained IOP elevation after IVI in eyes that received > 25 anti-VEGF injections. Furthermore, another study observed a reduced outflow facility in the injected eyes of patients who received more than 20 injections compared to no facility reduction in patients who received fewer than 10 injections. These changes in outflow facilities are thought to be due to a combination of mechanical causes, such as trabecular obstruction by microparticles from the injector, trabecular inflammation after IVI, and zonula disruption, but also due to a dysregulation of endogenous VEFG expression in the trabecular meshwork, which acts as a paracrine regulator of convectional outflow facility [12]. We cannot rule out that the average number of injections in our patients (23±12.4 IVIs) might have influenced the IOP increase, however, it has been reported more as a maintained IOP rather than an IOP spike in patients with > 25 IVI [12,13].

Another patient characteristic that may play a role is a preoperative elevated IOP. Patients with glaucoma tend to have a higher baseline IOP and due to their condition, they could take longer to return to baseline IOP after IVI [8,12,14,15]. However, information on short-term IOP following IVI in glaucoma patients is limited because clinical trials regarding anti-VEGF therapies tend to exclude eyes with this pathology.

Regarding axial length, hyperopic eyes may have a higher IOP elevation after IVI because of a relatively large volume injection into the smaller eye [16]. Some studies have suggested that IOP elevation, after intravitreal injection, tends to be greater in patients with phakic conditions [14]. Another factor that may affect IOP spike after IVI is previous pars plana vitrectomy. These patients were found to have a higher short-term risk of IOP elevation due to the absence of vitreous reflux when the needle was withdrawn [16]. Finally, ocular rigidity appears to affect the IOP. A positive correlation between ocular rigidity and a higher risk of IOP elevation after injection has been reported [17].

As mentioned above, elevations in IOP immediately after IVI are not uncommon, however, they are usually transient. Kim et al. reported that elevated IOP after IVI can return to values below 30 mmHg without any intervention [16]. However, there is no consensus in the literature regarding anterior chamber paracentesis use to normalize IOP. In most reported cases of increased IOP following IVI, the pressure returned to normal values with local conservative therapies (ocular massage and/or IOP-lowering drops). Differences in the management of IOP elevation after IVI may be due to differences in practice patterns, clinician experience, and clinical context.

Although aflibercept PFS could be associated with a higher IOP elevation risk, knowing injector characteristics may significantly decrease this risk. Furthermore, aflibercept PFS has several advantages over traditional self-filled IVI. PFS implies less manipulation of the medication, reduced total injection time, and decreased endophthalmitis rate [7,8]. Some studies have reported that silicone oil droplets decreased with ranibizumab PFS [18]. One may infer that silicone oil droplets may decrease with aflibercept PFS, as PFS has been subjected to an optimized siliconization process.

The fact OP incidence was higher than that reported by EMA (1.1 cases every 10.000 PSF versus 3.1 cases every 10.000) could be explained because although the IVI protocol was always followed, the thorough alignment of the plunger and force used during the injection could not be measured. On the other hand, it could be that some patients had a transitory non-symptomatic IOP after aflibercept PFS, being underdiagnosed as IOP was registered only in symptomatic patients. To avoid the risk of infection in our routine practice, we frequently do not check IOP if there are no symptoms. The lack of a retrospective control group is that we do not have registers of IOP incidence with vial preparations.

## Conclusion

In conclusion, although the use of aflibercept PFS has some advantages over traditional self-filled IVI, the increased risk of transient IOP elevation should be known and considered by specialists. A pool of patients may be at higher risk of developing IOP elevation after aflibercept PFS injection. Further studies with a control group are needed to define the ocular characteristics that might play a role in IOP elevation and to try to develop the ideal intravitreal syringe.
